# Supplementary material for: Gene Profiling of a 3D Psoriatic Skin Model Enriched in T Cells: Downregulation of PTPRM Promotes Keratinocyte Proliferation through Excessive ERK1/2 Signaling
Source: Cells. 2022 Sep 16;11(18):2904. doi: 10.3390/cells11182904 (PMC9497242; doi:10.3390/cells11182904)
Supplement: Supplementary file 1 [file cells-11-02904-s001.zip › cells-1860362-supplementary.pdf]

SUPPLEMENTARY MATERIAL

Supplementary Figures

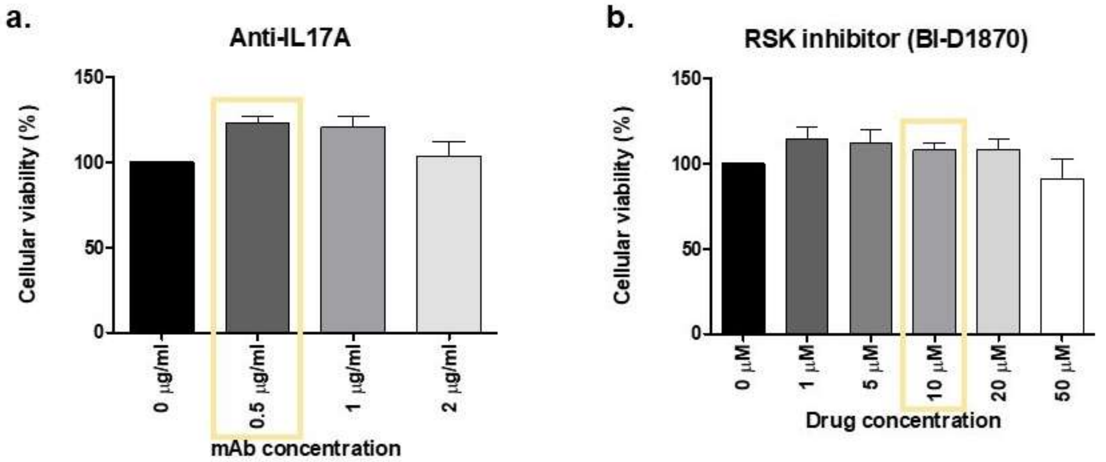

Supplementary Figure S1. MTS assay performed on psoriatic keratinocytes grown as monolayer to test three different doses of neutralizing anti-IL17A (0.5, 1 and 2 µg/ml) (a), and five different doses of ribosomal s6 kinase inhibitor (BI-D1870, 1, 5, 10, 20 and 50 µM) (b).

Supplementary Tables

Supplementary Table S1. Complete list of antibodies used for indirect immunofluorescence, EMSA and Western blot analyses.

Antigens	Biological source	Company	#Cat	#Lot	Dilution
Primary antibodies					
Anti-human PTPRM	Mouse monoclonal	Abcam	Ab233815	GR3241130-4	1:1000 overnight
NELL2	Rabbit monoclonal	Abcam	Ab181376	GR152681-1	1:1000
p-ERK1/2	Rabbit polyclonal	Cell Signaling Technology	9101S	31	1:1000
ERK1/2	Rabbit polyclonal	Cell Signaling Technology	9102S	27	1:1000
Anti-β actin	Mouse monoclonal IgG1	Abcam	A5441	I22M4782	1:30000
p-NF-κB	Mouse monoclonal	Santa Cruz Biotechnology	Sc136548	L3021	1:100
NF-κB	Mouse monoclonal	Santa Cruz Biotechnology	Sc8008	D1122	1:500
p-Sp1	Rabbit polyclonal	Abcam	Ab227383	GR3324683-13	1:500
Sp1	Rabbit polyclonal	Abcam	Ab59257	GR3404039-4	1:500
Anti-human Ki-67	Mouse monoclonal IgG_1,κ	BD biosciences	556003	8239549	1:400
Secondary antibodies					
Anti-mouse Alexa Fluor 488	Goat polyclonal IgG	Life	A11001	1890503	1:1500
Anti-rabbit HRP	Goat polyclonal IgG (H+L)	Jackson ImmunoResearch Laboratories Inc	111-035-003	I46270	1:60000
Anti-mouse HRP	Goat polyclonal IgG	Jackson ImmunoResearch Laboratories Inc	115-035-003	I46501	1:60000

Supplementary Table S2. Oligonucleotides used as labeled probes or competitors in the EMSAs.

Oligonucleotide	Top strand (5'-3')<br>Bottom strand (5'-3')
**Sp1**	GATCATATCTGCGGGGCGGGGCAGACACAG<br>GATCCTGTGTCTGCCCCGCCCCGCAGATAT
**NFKB**	GATCAGTTGAGGGGACTTTCCCAGGC<br>GATCGCCTGGGAAAGTCCCCTCAACT
**NFI**	TTATTTTGGATTGAAGCCAATATGAG<br>CTCATATTGGCTTCAATCCAAAATAA

Supplementary Table S3. Comparison between published microarray and RNA-seq datasets with 60 deregulated genes of our study.

Gene symbol	Gene name	Gudjonsson et al. [31]	Li et al. [32]	Pasquali et al. [3]	Our study	Our study
		L vs H FC	L vs H FC	PK vs HK FC	PS^{+T} vs HS^{+T} FC	PS^{+T} vs HS^{-T} FC
**KRT16**	Keratin, type I cytoskeletal 16	16.980	6.22	7.36	1.242	1.573
**DEFB4A**	Defensin Beta 4A	196.997	19913.19	32.82	0.826	6.538
**KYNU**	Kynureninase	25.465	23.37	20.43	1.099	1.652
**C10orf99**	Putative uncharacterized protein C10orf99	41.394	47.31	13.86	2.764	2.85
**SERPINA12**	Serpin A12	0.242	0.15	0.05	1.025	0.877
**SERPINA3**	Alpha-1-antichymotrypsin; Serpin peptidase inhibitor, member 3	2.222	2.27		1.217	2.443
**SERPINB9**	Serpin B9	2.098	2.50		1.394	2.784
**CXCL11**	C-X-C motif chemokine 11	2.097	6.10		0.273	2.208
**CXCL10**	C-X-C motif chemokine 10	7.053	11.54	2.20	0.304	2.880
**AQP9**	Aquaporin-9	0.306	0.13	0.15	0.314	0.288
**IL17RD**	Interleukin 17 receptor D	0.494	0.46		0.692	0.710
**IL17D**	Interleukin 17D	0.488	0.40		0.860	0.803
**LCE1B**	Late cornified envelope protein 1B	0.595	0.45	0.13	0.874	1.199
**CXCL9**	C-X-C motif chemokine 9	6.056	9.35		0.179	8.855
**MMP1**	Matrix metalloproteinase 1	3.270	20.19		1.902	6.531
**AKR1B10**	Aldo-keto reductase family 1 member B10	57.643	78.20	22.94	2.439	2.225
**FLG2**	Filaggrin-2	0.349	0.14	0.07	0.937	0.463
**UNC93A**	Protein unc-93 homolog A	2.710	2.64		1.539	0.791
**LEPR**	Leptin receptor	0.301	0.24		1.037	0.870
**LTB4R**	Leukotriene B4 receptor	2.268	2.82	2.83	1.523	1.722
**CCL27**	C-C motif chemokine 27	0.129	0.08	0.24	0.330	0.299
**HOXA10**	Homeobox A10	0.409	0.46		0.810	0.685
**SPRR2G**	Small proline-rich protein 2G	8.063	28.47		0.597	0.610
**SPRR4**	Small proline-rich protein 4	0.373	0.12	0.12	0.307	0.303
**LDLR**	Low density lipoprotein receptor	2.393	2.26	2.33	1.025	1.441
**TNS1**	Tensin 1	0.488	0.45		0.599	0.600
**HMGCS2**	Hydroxymethylglutaryl-CoA synthase, mitochondrial	0.444	0.14	0.38	0.870	0.830
**POSTN**	Periostin	0.385	0.34	0.05	1.408	1.814
**TGFBR3**	Transforming growth factor beta receptor 3	0.439	0.41	0.66	0.847	0.754
**WISP2**	WNT1-inducible-signaling pathway protein 2, WISP2 protein	0.463	0.27		1.536	1.386
**CDH3**	Cadherin 3	3.539	3.12	2.25	1.462	1.753
**EDIL3**	EGF-like repeat and discoidin I-like domain-containing protein 3	0.465	0.28	0.28	0.889	0.823
**AIM2**	Absent in melanoma 2	2.269	6.41		0.439	1.495
**CYP2C18**	Cytochrome P450 2C18	2.148	1.83		0.580	0.739
**ARG1**	Arginase-1	3.665	5.39		1.541	1.839
**GJB2**	Gap junction beta-2 protein	7.311	18.82	9.32	1.648	2.384
**CXCR4**	C-X-C chemokine receptor 4	3.081	3.32	2.17	0.610	1.541
**CREB5**	CAMP responsive element binding protein 5	0.495	0.49		0.750	0.841
**CST6**	Cystatin-M	0.343	0.16	0.46	0.716	0.501
**HMOX1**	Heme oxygenase 1	2.174	2.34		0.779	0.815
**CD1A**	Cluster of differentiation 1a	0.509	0.40		0.207	0.363
**CYP4B1**	Cytochrome P450 family 4 subfamily B member 1	0.426	0.25		0.344	0.294
**CYP2J2**	Cytochrome P450 family 2 subfamily J member 2	0.501	0.25	0.47	0.571	0.665
**CD36**	Cluster of differentiation 36	2.091	2.53	6.86	2.091	1.866
**FABP5**	Fatty acid binding protein 5	2.756	10.45	7.52	2.055	1.909
**PTPRM**	Receptor-type tyrosine-protein phosphatase mu		0.60	0.41	0.408	0.327
**NR2F2**	Nuclear receptor subfamily 2 group f member 2		0.56		0.200	0.259
**MCHR1**	Melanin concentrating hormone receptor 1		1.67		3.053	2.989
**CCL3**	C-C motif chemokine 3		9.21		0.835	1.710
**ELL2**	Elongation factor for RNA polymerase II		1.72	1.53	2.142	2.723
**IL1R2**	Interleukin 1 receptor type 2		0.77		0.231	0.502
**IL1A**	Interleukin 1 alpha		0.70		0.440	0.592
**IL12RB2**	Interleukin 12 receptor subunit beta 2		3.66	2.15	1.369	1.550
**IL18**	Interleukin 18		0.61		0.501	0.471
**CAMP**	Cathelicidin antimicrobial peptide		7.70		1.902	2.822
**FABP6**	Fatty acid binding protein 6		1.96		3.677	3.229
**CCR10**	C-C chemokine receptor 10		0.43		0.338	0.487
**POU2AF1**	POU class 2 homeobox associating factor 1		1.23		4.451	4.262
**KRT31**	Keratin 31		0.27	0.37	0.130	0.253
**TRPS1**	Transcriptional Repressor GATA binding 1		0.89	1.50	2.152	4.122

Genes whose fold change are indicated in black are similarly deregulated (either repressed or activated) whereas those in red are regulated in opposite ways between our study and those of Gudjonsson et al. [31], Li et al. [32] or Pasquali et al. [3]. L: lesional psoriatic skin; H: healthy skin; PK: psoriatic keratinocyte; HK: healthy keratinocytes. PS: psoriatic skin substitutes; HS: healthy skin substitutes; T: T cells; FC: fold change.
